# Characterization of Sweat Drying Performance of Single Layered Thermal Protective Fabrics Used in High-Risk Sector Workers’ Clothing

**DOI:** 10.3390/polym14245393

**Published:** 2022-12-09

**Authors:** Sumit Mandal, Ishmam Zahin Chowdhury, Nur-Us-Shafa Mazumder, Robert J. Agnew, Lynn M. Boorady

**Affiliations:** 1 Department of Design and Merchandising, Oklahoma State University, Stillwater, OK 74078-5061, USA; 2 Textile Protection and Comfort Center, Wilson College of Textiles, NC State University, Raleigh, NC 27606-3700, USA; 3 Fire Protection and Safety Engineering Technology Program, Oklahoma State University, Stillwater, OK 74078-5061, USA

**Keywords:** fire protective fabrics, sweat moisture, moisture management, sweat drying performance, thermo-physiological comfort, technical textiles

## Abstract

Absorption and transportation of moisture from sweat are the crucial properties of the fabrics used in performance clothing. Sweat moisture is a significant factor that may cause discomfort to the wearer. The majority of the injuries and fatalities that happen to the high-risk sector workers in their line of duty may be caused by inadequate comfort provided by the protective uniform. The purpose of this study is to scientifically investigate the sweat drying performance of the different protective fabrics used in high-risk sectors’ workers’ clothing. Firstly, this study experimentally analyzed the sweat drying of protective fabrics with different attributes under various ambient environments and wearers’ internal physiology. Secondly, this study explained the phenomena of sweat drying in protective fabric through the theory of heat and mass transfer. Sweat drying performance of the fabrics used in functional clothing mainly depends on the evaporative resistance regardless of the presence of water and oil repellent coating on the fabric surface. The drying performance increases with the increased wetted area and increased air flow. The wetted area depends on the absorption and wicking properties of the fabrics. The findings of this research will advance the field by developing knowledge on sweat drying performance of fabrics used in protective clothing; in turn, this could provide better comfort and safety to high-risk sectors’ workers.

## 1. Introduction

In 2019, the National Fire Protection Association (NFPA) reported 9 firefighter fatalities in more than 50,000 wildfire-incidents in the USA [1,2,3,4]. In particular, Oklahoma is one of the 10 most wildfire-prone states of the USA (as per 2018 statistics from the Insurance Information Institute, USA), and wildfire causes numerous heat stress-related injuries to our firefighters [5,6]. Additionally, more than 1600 oilfield workers died from injuries in the oil-and-gas drilling industry from 2008 through 2017. Between 2017−2019, the annual number of fatalities increased from 8 to 22 in the oil-fields [7,8]. While being the 6th in oil production, Oklahoma was documented as one of the top three injury prone states for oil-field workers [9,10,11]. Notably, the majority of these fatalities and injuries results from inadequate comfort provided by thermal protective fabrics used in firefighters’ clothing [12,13].

In the high-risk sectors’ working scenario, a worker needs to complete arduous work in long shifts typically in steep terrain and/or at high elevations [14,15]. Additionally, the working environment of firefighters is usually hot and dry and the presence of fire increases the exposure to heat [16,17]. In general, skin and core temperature of human beings is 32 °C and 37 °C, respectively, at normal ambient temperature; however, in the case of high-risk workers, their skin and core temperature start to increase when they perform hard work in a hot environment. When the skin and core temperature start to increase, workers bodies start to regulate the heat by using the mechanism of vasodilation, where a widening of blood vessel occurs near the surface of the skin and that leads to increased blood flow with flushing or warmth. At the skin temperature of 37 °C, the sweat glands activate near the surface of the skin and produces sweat. As the sweat evaporates, the cooling of the body occurs to workers. This situation provides better thermo-physiological comfort to workers [18,19,20].

In order to provide a better working environment for high-risk workers, it is necessary that fabrics used in workers’ clothing should not accumulate sweat. In turn, the fabric should dry as soon as possible to provide better thermo-physiological comfort to wearers [19,21,22,23]. Drying mechanisms of fabric refers to the diffusion of sweat into vapor, i.e., evaporation out into air. Scientifically, the fabric is a porous media; so, the sweat water molecules in a fabric are loosely and non-statically H-bonded to each other and/or to the fabric in liquid form. During drying, the sweat water molecules break their bonds to each other as well as to fabric and then, evaporates into the air. In general, breaking of the sweat water molecules and their evaporation depends upon the temperature of the sweat moisture in the fabric, the humidity of the ambient air and the flow rate of ambient air. As human beings start to sweat when their skin temperature is nearly equivalent to their core temperature, the temperature of the sweat water is usually less than or equal to 37 °C. Even though the temperature of this sweat water is much lower than the temperature of boiling water (i.e., 100 °C), this temperature could facilitate the process of sweat water diffusion to air. When the water molecules have enough energy to break the in-between H-bonds and surface tension, it may diffuse into the air. This evaporation of sweat water also depends upon the humidity of the ambient air. At high humidity, concentration of water molecules in the surrounding ambient air is very high. In this situation, the diffused water molecules could condense with the water molecules in the ambient air and may stick on the fabric; this phenomenon actually slows the drying process of fabric. Although temperature of the sweat water and humidity of the ambient air influence the diffusion of the water molecules towards air, this diffusion is a very slow process. It has been found that the diffused water molecules mainly stay on the outer surface of the fabric, bump into the fabric, and slow down the drying process. However, if the ambient air starts to flow, the attached water molecules start to evaporate very quickly and this evaporation process speeds-up if the ambient air flowing over the fabric has lower humidity than the humidity of the displacing air on the surface of the fabric [24].

Considering the above situation, a great deal of researchers investigated the drying mechanism of textile fabrics. Heinisch, et al. [25] mentioned that water molecules in the fabric could be loosely bound between the yarns used in the fabric or strong bounded by a hydrogen bond with the fibers used in the yarns. Drying mechanism of textile fabrics includes the removal of loosely/strongly bounded water and water content as well as the temperature of the fabrics; both play major roles in the drying of the fabrics [26]. During the constant drying of the fabric, theoretically the temperature of the fabrics are constant and unbound water content in the fabric linearly decreases depending upon the convective mass transfer coefficient between the wet fabric and ambient air [27]. After the removal of unbound water content, the bounded water in the fabric starts to leave through evaporation and at this stage the temperature of the fabric increases rapidly. It has been found that the evaporation of water occurs throughout the drying process and the water content in the fabric decreases non-linearly over time [28]. Notably, temperature of the fabric rises slowly when a fabric contains liquid water; however, this temperature rise occurs very rapidly when all water molecules have evaporated. Notably, the drying rate of a fabric is significantly lower than the water absorption rate of that fabric and drying rate is mainly dependent upon the types of fabrics used in the clothing [29,30].

Contextually, it has been found that different fibers, yarns and structural construction in the fabric could lead towards different levels of drying in certain time frames. It has been found that textile fabrics with different fibers, yarns and structural constructions could have different level of water absorption, wicking and water vapor transmission properties and these properties significantly affect the drying rate of the fabric. Gurudatt, Nadkarni and Khilar [29] mentioned that drying rate is independent upon the fiber types during the constant drying of the fabric; after the constant drying, fiber types and absorbed water volume play a major role to rapidly increase the temperature of the fabric. Changing in the structure of the fabric could also change the drying rate of a fabric. Actually, changing the structure of the fabric affects its absorption and spreading of water in the fabric, which ultimately affects the sweat evaporation of the fabric to dry. While drying, the level of hygroscopicity of the constituent fabric materials has high significance in dynamic moisture transfer. A facilitated mass and energy transfer has been observed for highly hygroscopic fibers [31]. Das, et al. [32] has mentioned that the water vapor diffusion mainly depends on the porosity of the fabrics, which further facilitates via fabrics’ wicking properties.

Although previous researchers investigated the drying rate of the fabric, it is evident that most of these studies focused on drying of the fabric during wet processing such as dyeing and finishing. A very limited amount of studies focused on sweat drying in consideration with the wearers’ thermo-physiological comfort [19,23]. Additionally, drying of fabrics were mainly studied in consideration with the ambient environment (e.g., air speed, humidity) or fabric attributes (e.g., fibers, yarns and structural construction). To date, the authors have found no research that has experimentally analyzed the holistic impact of ambient environment, fabric attributes and wearers internal physiology such as sweat volume on drying of fabrics. Additionally, previous studies mainly focused on the drying of fabrics used in regular clothing; none of these studies focused on drying of protective fabrics used in high-risk sectors’ workers clothing. Additionally, no studies explained the phenomena of drying in protective fabrics based on the theory of heat and mass transfer.

This study will scientifically investigate sweat drying of the different protective fabrics used in high-risk sectors’ workers clothing. For this, firstly, this study will experimentally analyze the sweat drying of protective fabrics under different fabric attributes, ambient environment, and wearers’ internal physiology. Secondly, this study will explain the phenomena of sweat drying in protective fabric through the theory of heat and mass transfer. This is important to study because high-risk sectors’ workers sweat a lot and it could significantly wet fabric used in their clothing. This situation will lower the thermo-physiological comfort of wearers and may lower their working performance or cause potential injuries due to heat stress or strain. This study will advance the field of protective textiles and clothing by developing knowledge related to the sweat drying performance of fabrics used in high-risk sector workers ‘clothing. Overall, the novelty of this study lies in systematic understanding of the drying of fabrics that could help textile/materials scientist to develop new fabrics for better working safety of high-risk sectors’ workers such as firefighters, oil-gas industry workers.

## 2. Materials and Methods

In order to fulfill the first objectives, we evaluated the physical properties and sweat drying performance of fabrics used in high-risk sector workers’ clothing. The association between fabric properties and sweat drying performance under different ambient environment and wearers’ internal physiology are discussed in order to identify the key fabric properties affecting the performance. For fulfilling the second objectives, we reviewed the theory of heat and mass transfer in porous media and then applied in the context of the drying for protective fabrics.

### 2.1. Fabric Selection and Properties Evaluation

**Figure 1:**
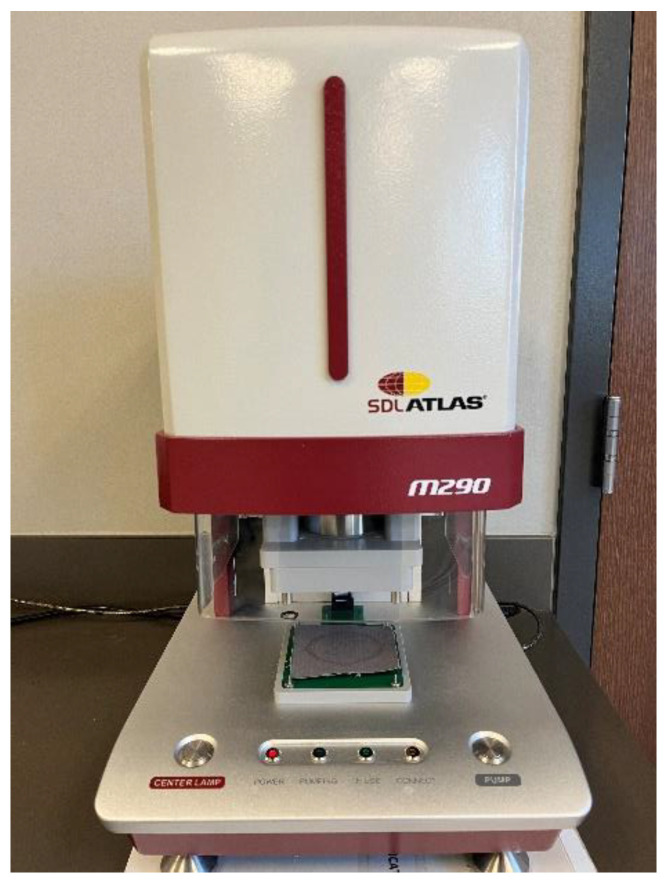
Moisture Management Tester as per AATCC 195 method.

In this study, we selected four different thermal protective fabrics that are commercially used to manufacture high-risk sector workers’ clothing. These fabrics comprise of different fibers and constructions in terms of Ends Per Inch (EPI) and Picks Per Inch (PPI). The physical properties of these fabrics were measured by using various American Society for Testing Materials (ASTM) standards (Table 1). The behavior of the sweat moisture on the fabric was measured in our Textile and Apparel Science Laboratory (TASL) at Oklahoma State University (OSU) by using the Moisture Management Tester, SDL Atlas, Rock Hill, SC (Figure 1) as per American Association for Textile Chemists and Colorists (AATCC) 195 test method. The behavior of the moisture on fabrics were measured in terms of Wetting Time for top and bottom surfaces (WT), Absorption Rate for top and bottom surfaces (AR), Max Wetted Radius for top and bottom surfaces (MWR), Spreading Speed top and bottom surfaces (SS), and Accumulative One-Way Transport Capacity (AOTC) (Table 2). Finally, Overall Moisture Management Capacity (OMMC) of the fabrics were also calculated based on Equation (1) and the OMMC values are presented in Table 2. Measurement of such units have been extensively described by Yao, et al. [33].

### 2.2. Sweat Drying Performance Evaluation

In order to evaluate the sweat drying performance of the above selected fabrics, 3 sample specimens (38 × 38 cm^2^) from each fabric were prepared. Then, the sample specimens were conditioned under standard atmospheric environment, i.e., temperature at 21 °C and relative humidity of 65% as per ASTM D 1776 standard.

**Figure 2:**
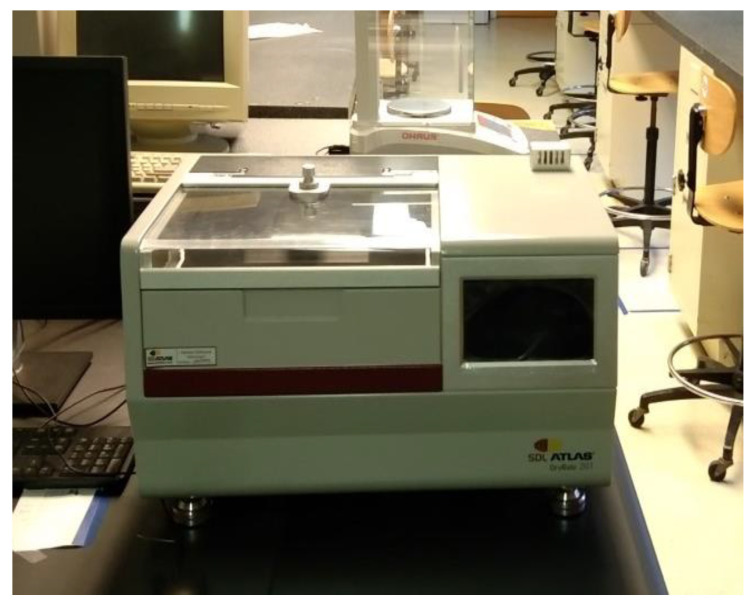
Fabric Drying Performance Evaluation Tester, SDL Atlas, Rock Hill, SC as per AATCC 201 method.

After conditioning, the sweat drying performance of the fabric specimens were measured based on the evaporative rate of the specimens. Here, the drying performance was measured using equipment in our TASL that works according to AATCC 201 method (Figure 2). This test was performed under controlled laboratory temperature (21 °C) and relative humidity (65%) conditions. The equipment determined the drying rate of the fabric specimens exposed to a prescribed volume of deionized water while in contact with a heated plate set at 37 °C. Deionized water (18.2 MΩ-cm) was prepared using a Labconco WaterPro PS polishing system, Kansas City, MO. The temperature of the heated metal plate was simulating the human skin surface temperature at which a human body starts to perspire. At first, according to AATCC 201 method, drying performances of the selected specimen were evaluated under 0.2 mL sweat volume while maintaining 1.5 m/s airflow across the width of the specimen. Additionally, drying performance were measured under different amount of sweat volume such as 0.5, 1, 1.5 mL. The ambient air flow across the width of the specimen were controlled at 0.5, 1, and 1.5 m/s for every sweat volume. As per methodology, this research considered the sweat-drying performance of the fabric material used in high-risk sector work wear in non-hazardous environmental conditions. Even though such methodology did not address the high temperature of the wildland firefighting and oil-and-gas fields, the approached methodology let us address the comparative drying performances of the correspondent fabric materials. The environment in the drying chamber is controlled. Airflow and sweat moisture amounts have been changed to consider the environmental and personnel variability in such working environments. Air flow has been maintained 0.5–1.5 m/s to consider the variability in air speed while maintaining the similarity with the ASTM D737. To effectively simulate the sweat drying from skin, 37 °C skin temperature was simulated.

During actual testing, firstly, we turned on the temperature controller for the heater and fan to let the metal plate temperature stabilize to 37 ± 1 °C. Then, we placed the fabric specimen on the heated plate for 5 min to allow the specimen to equilibrate to the metal plate temperature with the side of the specimen intended to be next to the skin placed against the metal plate. A metal strip was placed on the edge of the specimen that is closest to the air fan to the metal plate surface. Then, we set the ambient air speed and sweat volume as per the requirement of the test and started the test. At this stage, we also checked the wicking profile generated by the sweat water. If the water wicked the edge of the specimen, we used the specimen with larger size. The temperature of the fabric specimen was collected and recorded via thermocouple every 0.2 s until the temperature of the fabric reached to the initial temperature. When the fabric temperature reached to the initial temperature, it was called the end time of the test. In this study, the sweat drying rate was calculated based on Equation (1), where R = Drying Rate (mL/h), V = Sweat Volume used in the Test, and Drying Time = Test End Time − Test Start Time.

(1) $$R=\frac{V}{\mathrm{Drying}\;\mathrm{Time}}$$

### 2.3. Procedure for Data Analysis

The properties (Table 3) and sweat drying performance values of all the fabrics (Table 4) were statistically normalized and t-tests were carried out using SPSS software version 26, IBM, Armonk, NY. The associations between fabric systems’ properties and sweat drying performance values were inferred based on the sign (+ or −) of the T-stat values obtained from the *t*-tests. *p*-values obtained from the t-tests for each fabric property were also analyzed. For the *p* < 0.05, the property was identified as the key affecting factor for sweat drying performance. The relationship between fabric properties and sweat drying performance values was plotted and coefficients of determination (R^2^) of these plots were calculated. A R^2^ value with proximity to 1 for a particular plot was inferred as a strong association between the respective fabric property and sweat drying performance. Furthermore, inference tests [hypothesis test (*p*-value) and 95% confidence interval (upper and lower limit)] were carried out to understand the differences in sweat performance between two types of fabric systems. Finally, the sweat drying mechanisms of fabrics depending upon the sweat volume and ambient air flow were analytically and mathematically modeled based on the test configuration shown in Figure 1. Analytically, heat and mass transfer during the testing were identified and a few basic assumptions were made regarding the sweat drying. For the mathematical modeling, the fundamental theory of heat and mass transfer through solid or porous substances was extensively applied.

## 3. Results and Discussion

### 3.1. Experimental Analysis of Sweat Drying Performance of Protective Fabrics under Standard Condition

As per the AATCC 201 standard, the sweat drying performances of the selected fabric samples (Table 1) are tabulated in Table 3.

From the table, it is evident that sample (c) had a lower drying rate compared to (a), (b), and (d). This could be attributed to the significantly higher evaporative resistance of sample (c). Evaporative resistance refers to the moisture vapor permeability and wicking properties of textile fabrics. Hence, with higher evaporative resistance, the fabric may not quickly transfer water from one side to the other sides [22]. However, the difference in the drying rates of (a), (b) and (d) could not actually be addressed via the evaporative resistance of corresponding samples. The evaporative resistances of these samples varied a little, which were also similar in the case of drying rates. The other physical properties of these samples varied but could not be related to the resultant drying rates. Hence, to further investigate the sweat drying rate performance, the moisture management properties of such samples were addressed. As evaporation through the fabric is the subsequent step of wetting and wicking of absorbed sweat, moisture management properties could have a significant or insignificant effect on the drying performance.

**Figure 3:**
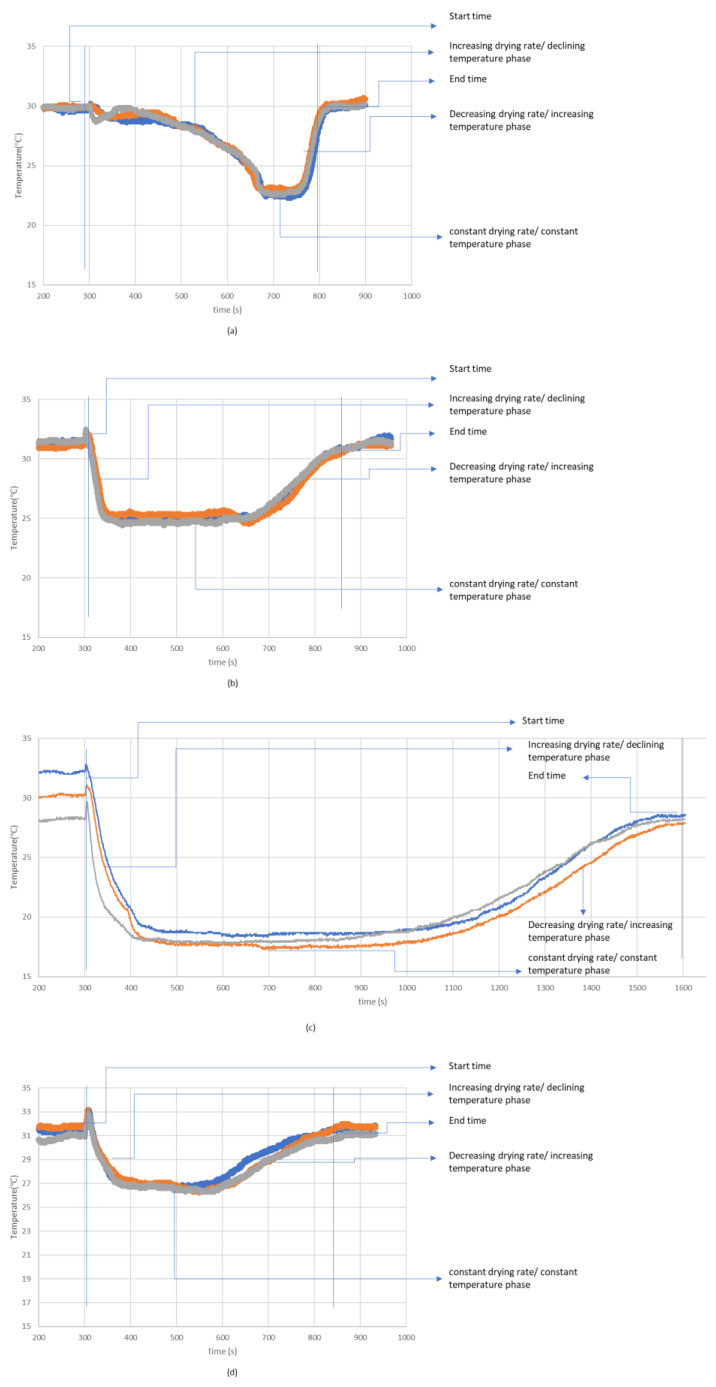
Temperature time plotting for drying rates of different samples (**a**–**d**). Each color identifies different trials of the same samples.

From the moisture management properties (Table 2), sample (a) could be identified as somewhat water resistant due to the high negative value of AOTC. AOTC refers to the difference in moisture content between the two opposite surfaces for a given time. A high negative value of AOTC referred to the very slow transport of sweat from the application side to the opposite. This phenomenon is also evident in the temperature time plotting generated while the fabric is drying (Figure 3). In the temperature-time graphs, fabric (a) did not see any significant temperature change for some time. Due to the high negative value of AOTC, sweat transportation from the inner to the outer layer took much of the drying process time. After the delayed sweat transportation to the outer surface, the temperature starts to fall. This identifies the availability of sweat on the evaporation surface and the gradual increase in sweat evaporation. This phase could be referred to as the ‘increasing drying rate’ or the ‘declining temperature phase [34].’ Due to less sweat transporting to the evaporation surface, sample (a) had observed this phase longer than other samples.

Later, when the evaporation surface was saturated with the sweat amount, an equilibrium was achieved between heat transfer and evaporation. This could be identified as the ‘constant temperature phase’ in the temperature-time plotting. According to Lyons and Vollers [34], this phase can also be considered a ‘constant drying rate’ period. Here, the same amount of sweat evaporation absorbed the same amount of heat from the surroundings, maintaining the constant temperature. When the evaporation surface became less saturated, less availability of sweat for evaporation caused the temperature to rise. The drying rate started to fall, which can be identified as the ‘declining drying rate’ phase for the clothing. Lyons and Vollers [34] mentioned that the evaporation plane moves into the fabric in such a phase. Considering the low evaporative resistance of sample (a), facilitated evaporation could occur within the fabric at this stage.

Unlike sample (a), samples (b), (c), and (d) easily transferred moisture to the evaporative surface. Positive values of AOTC indicate that sweat could be easily transferred from the inner to the outer layer. Very small duration of ‘increasing drying rate’ phases had been observed for these samples. As a result, saturation of evaporation surface was achieved easily; ‘constant drying rate’ was achieved early in the drying phases. Significantly longer ‘constant drying rate’ phases were observed for such samples. For samples (b) and (d), positive AOTC could facilitate the wicking phase of drying phenomena. The high absorption rate on both sides of such samples could facilitate the wetting of the fabric surface. Both (b) and (d) achieved the highest maximum wetted radius in AATCC 195 test method. Hence, it can be inferred that such samples’ sweat absorption area on the evaporation side is higher than the sample (c). As the wetted area on the evaporation side is larger for (b) and (d), evaporation started to happen from a larger area. This leads to the evaporation of more sweat from a larger sweat-saturated area. In other words, the ‘constant drying rate’ phase had been achieved in a larger area. Hence, high drying rates were achieved for samples (b) and (d). Additionally, due to less evaporative resistance, the ‘declining drying rate’ phase was facilitated similar to sample (a).

For sample (c), even though the saturated evaporation surface is achieved quickly, the drying rate is less compared to the other specimen. Sample (c) had a very high AOTC value. This identifies the fastest transfer of moisture or sweat to the evaporation surface. However, the max wetted radius of sample (c) was less than the sample (b) and (d). This data indicated a very high concentration of sweat in the sweat-saturated evaporation surface in the dry-rate testing. As the same amounts of moisture were applied on (b), (c), and (d), sample (c) has high sweat amount per unit evaporation area compared to samples (b) and (d). Hence, a high amount of sweat was being evaporated from per unit evaporation area (considering the adjacent area of the thermocouple) for sample (c). As a result, sample (c) achieved the ‘constant drying rate’ at a lower temperature than samples (b) and (d). However, considering the longer duration of ‘constant drying rate’ compared to samples (b) and (d), it can be assumed that less amount of sweat was being evaporated from the small sweat-saturated area. In the declining ‘drying rate phase,’ the evaporation plane moves into the fabric. Due to significantly higher evaporative resistance, a slow ‘declining drying rate’ phase was observed.

From the above discussion, we can infer that fabric moisture management is closely related to the temperature profile of sweat-drying fabrics. To relate the moisture management properties with the drying performance, we need to consider fabric (a) as an outlier. For being water-resistant and breathable, sweat evaporation could occur even before the sweat absorption by sample (a). For samples (b), (c), and (d), sweat drying performance could be related to moisture management properties. Moisture management properties of such samples represent both the cross-sectional and surface transport of sweat, which could be equivalent to the wicking and wetting of such samples, respectively. From the temperature-time plotting, it can be inferred that a higher accumulative one-way transport index (AOTC) facilitates achieving the ‘constant drying rate’ phase. In the drying rate test setting, higher wicking is the simulant of such a scenario. Additionally, the absorption rate of surfaces from the MMT testing could be a simulant of the wetting phenomenon in drying rate testing. High absorption rates could also facilitate achieving a higher wetted radius, exposing more sweat to the evaporation front. Hence, with higher absorption speed and AOTC, sweat will be readily available on the evaporation surfaces, facilitating more sweat being exposed for evaporation. From the above discussion, three points could be inferred: firstly, higher values of AOTC facilitate achieving a quick ‘constant drying rate’ phase. It could also indicate more concentration of sweat in the unit evaporation area. Secondly, higher absorption speed and wetted radius could indicate a larger evaporation front, leading to more sweat evaporating. Thirdly, higher evaporative resistance could minimize the evaporation in the ‘declining drying rate’ phase. It could also affect the ‘constant drying rate’ phase, considering that evaporative resistance also refers to the moisture vapor permeability and wicking properties of textile substrate.

### 3.2. Experimental Analysis of the Sweat Drying of Protective Fabrics under Different Ambient Conditions

Following the inferences made in the previous section, different ambient conditions were addressed to evaluate their corresponding impact on the drying performance. Different sweat amounts and airflow were applied to simulate the different sweat-drying scenarios. Based on the findings from such test conditions, significant parameters affecting the drying performance have been addressed. Table 4 charted the average drying rate under different sweat drying conditions. The following sections will evaluate their effects.

#### 3.2.1. Sweat Drying Performance of Protective Fabrics at 0.5 m/s Ambient Air Speed

**Figure 4:**
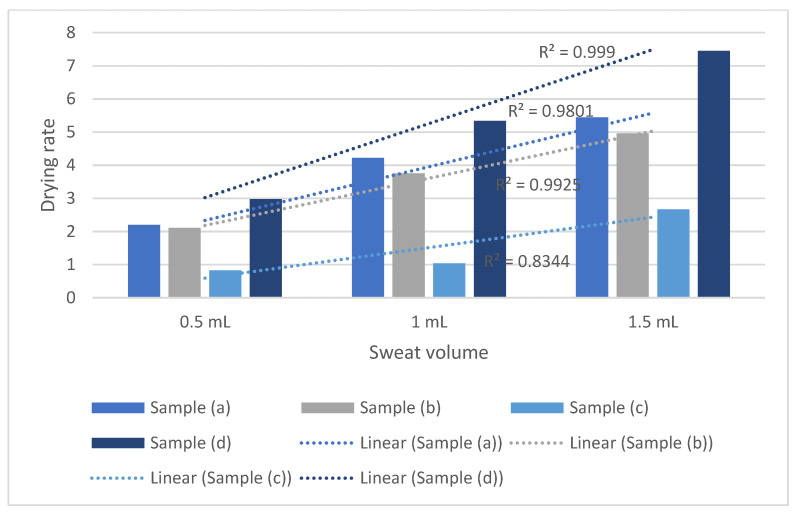
Sweat drying performance of protective fabrics at 0.5 m/s ambient air speed.

The sweat drying performance of the selected fabrics for 0.5 m/s ambient air speed has been charted in Figure 4. For every sweat amount, fabric samples (c) and (d) show the lowest and highest drying performance, respectively. Samples (a) and (b) have the second and third highest drying performance. All the samples saw increased sweat drying performance with the increase in sweat volume. Regression analyses were done to correlate the impact of sweat drying performance with the given sweat amount. How each sample was affected has been analyzed. The resultant regression equations between sweat volume and drying rate are: y_a_ = 1.62x + 0.7133, y_b_ = 1.425x + 0.7567, y_c_ = 0.92x − 0.3267, and y_d_= 2.235x + 0.7867; where y refers to the drying rate of corresponding fabric samples and x refers to the sweat amount. For such regression models, samples 1, 2, and 4 have R^2^ values > 0.95, which attributes to a high correlation between sweat volume and sweat drying performance. Sample 3 has an R^2^ value of 0.8344, indicating a greater than 80% variability could be addressed via such a regression model.

The regression coefficients for fabric samples (a), (b), (c) and (d) are 1.62, 1.425, 0.92 and 2.235, respectively. Hence, it is evident that the impact of sweat volume on the drying rate is significantly lower for sample (c) compared to the other three. This could be attributed to the higher evaporative resistance of sample (c). Evaporative resistance can be identified as a combined attribute of fluid permeability and wicking in clothing. The significance of such a parameter is that it also considers the presence of coating over textile substrates. Sample (d) saw the highest increase in the drying rate with the increase in the sweat volume. Sample (d) had the lowest evaporative resistance and cotton composition, which is more hygroscopic than meta/ para-aramid. Being more hygroscopic, cotton could contribute to facilitated absorption and wicking of sweat.

Samples (a) and (b) had similar tendencies of drying rate increase under different sweat volumes. Sample (b)’s evaporative resistance was slightly lower than the sample (a). However, the meta-aramid portion of sample (a) was much higher than the sample (b). Sample (b) had para-aramid in its composition, which was less hygroscopic than the meta-aramid. This could be why the drying rate of sample (b) saw less increase in drying rate with the rise of sweat volume. Similar to such, the different magnitude of increase in the regression coefficients between sample (a) and sample (d), as well as samples (b) and (d), could be more related to the hygroscopic portion rather than the evaporative resistance (no significant difference in between). However, samples (b) and (c) have similar compositions, where the higher evaporative resistance of the sample reduces the drying performance. Hence, it could be inferred that reducing the evaporative resistance could enhance the sweat drying performance of the fabric. Increasing the hygroscopic portion in the fabric composition could further enhance the magnitude of drying performance.

From the above discussion, it is already evident that increasing sweat volume increases sweat drying rate. The significant increase in the sweat drying performance due to the sweat volume could be either attributed to higher sweat concentration (g/cm^2^) [29] or larger evaporation front (wetted area), or a combination of both. As previously mentioned, the drying of textile substrates commonly follows three distinct phases: increasing drying rate (for reaching equilibrium with temperature and humidity), constant drying rate, and declining drying rate phase. The ‘constant drying rate’ phase is achieved when the fabric moisture content is above the fiber saturation point [35]. As the fabric surface achieves saturation, maximum possible sweat/ moisture is present in the evaporation front. The drying rates will even be approximately equal for different fabric types at this stage for such saturation [29]. Evaporation of sweat from the fabrics’ outer surfaces only depends on the humidity (diffusion) gradient at this stage. Hence, According to Gurudatt, Nadkarni and Khilar [29], higher moisture (sweat) concentration affects the declining phase rather than the ‘constant drying rate’ phase, as higher sweat concentration facilitates moisture movement to the interface. However, in the present study setting, it could also affect the ‘increasing drying rate’ phase as this phase also indicates the moisture or sweat movement to the evaporation interfaces due to per unit moisture/ sweat availability. To increase the evaporation in the ‘constant drying rate’ phase, saturation needed to be achieved from a larger area. The larger wetted area on the evaporation surface provides a larger evaporation front. More water being present in the evaporation front increased the sweat evaporation.

#### 3.2.2. Sweat Drying Performance of Protective Fabrics at 1 m/s Ambient Air Speed

**Figure 5:**
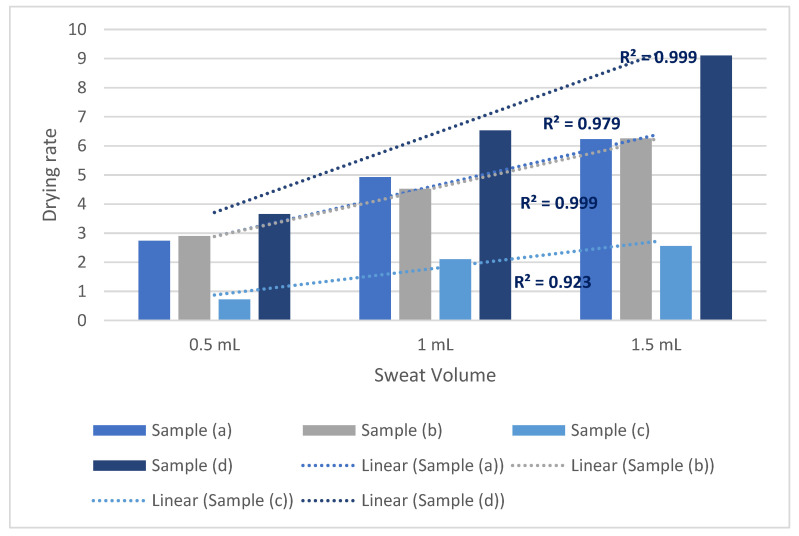
Sweat drying performance of protective fabrics at 1.0 m/s ambient air speed.

Similar to the 0.50 m/s, for airspeed 1.0 m/s, samples showed a somewhat similar tendency in drying rate performance (Figure 5). The regression equations between sweat volume and drying rate were as follows, y_a_ = 1.745x + 1.14, y_b_ = 1.675x + 1.2067, y_c_ = 0.92x − 0.0467, and y_d_= 2.72x + 0.99; here y refers to the corresponding drying rate and x refers to the sweat volume. Sample (d) saw the highest increase, and sample (c) saw the lowest increase in the drying rate with the increasing sweat amount. Aside from sample (c), sample (a), (b), and (d) has regression coefficients >1.5. Fabric sample (c) has a regression coefficient of 0.92. The coefficient of determination of such regression models is >0.90. Similar to the previous section, the lower values of drying rate for sample (c) were due to the high evaporative resistance. For samples (a), (b), and (d), the difference in such tendency is due to the more hygroscopic portion of samples. Sample (a) and sample (b) saw similar effects on drying rate due to the increase in the sweat volume. For sample (a), it was slightly larger (regression coefficients of 1.745 and 1.675) than the sample (b) due to the higher meta-aramid portion in the composition.

#### 3.2.3. Sweat Drying Performance of Protective Fabrics at 1.5 m/s Ambient Air Speed

**Figure 6:**
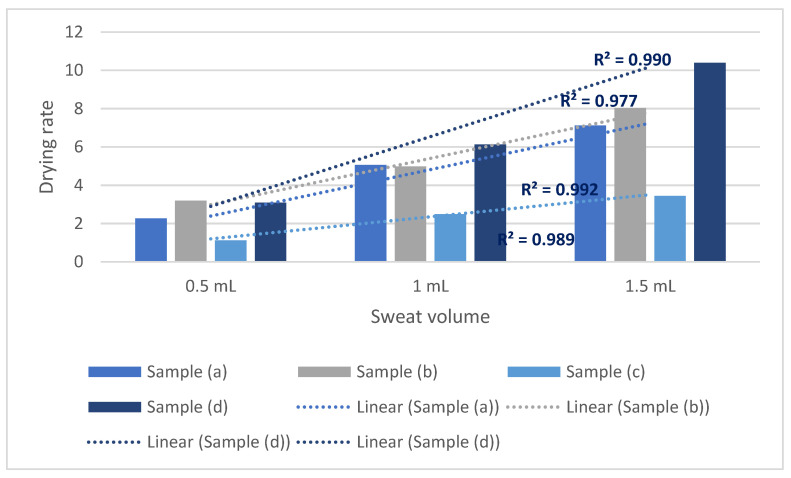
Sweat drying performance of protective fabrics at 1.5 m/s ambient air speed.

For 1.5 m/s airspeed, the regression equations between sweat volume and drying rate are as follows, y_a_ = 2.425x − 0.0333, y_b_ = 2.415x + 0.5733, y_c_ = 1.155x + 0.0433, and y_d_ = 3.645x − 0.75; here y refers to the corresponding drying rate and x refers to the sweat volume. Samples (a), (b), (c), and (d) have regression coefficients of 2.425, 2.415, 1.155, and 3.645 with the coefficients of determination > 0.95 (Figure 6). In this airflow condition, the impact of sweat volume is much more prominent for sample (c) compared to the previous airflow conditions. However, sample (c) still saw the lowest impact due to the increased sweat volume. Hence, similar to the previous sections, the effects of sweat volume on the drying rate performance can be attributed to the corresponding evaporative resistance and hygroscopic composition. Hygroscopic composition facilitates absorption. This could lead to having more sweat-wetting area on the evaporation front, leading to more sweat drying. The slight difference in the impacts of sweat volume on drying rate performance for samples (a), (b), and (d) can be attributed to this.

#### 3.2.4. Sweat Drying Performance of Protective Fabrics at Different Sweat Volume at a Critical Ambient Air Speed

**Figure 7:**
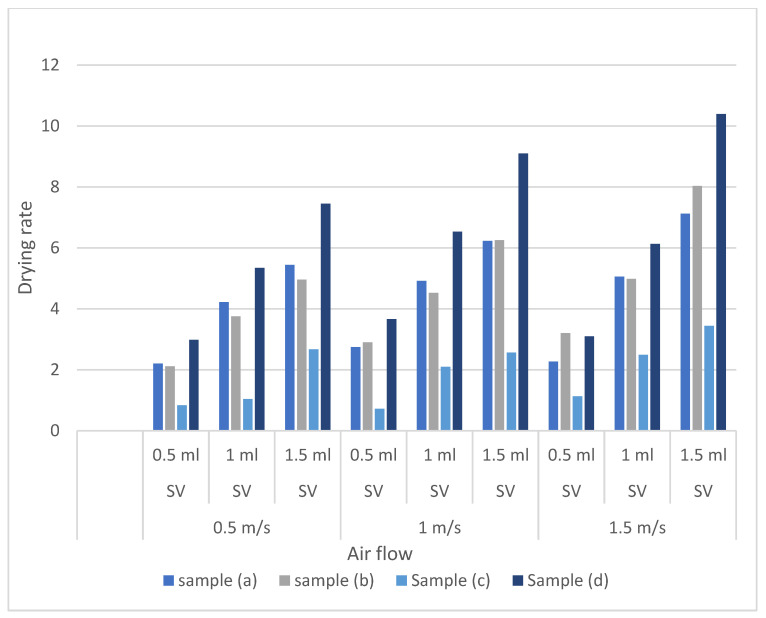
Sweat drying rate under different ambient condition.

Based on the previous discussions, it is evident that airspeed also increased the drying rate. From Figure 7, we can infer that the effect of airflow is more evident in higher sweat volume. However, the magnitude of airflow impact is not as prominent as the sweat volume. The effect of airflow on the drying rate could only be related to maintaining the humidity gradient over the evaporation front. Airflow constantly removed the saturated air layer from the evaporation front. As a result, the diffusion gradient over the evaporation front was always maintained. With the higher airflow, a higher evaporation gradient was present over the evaporation front. Such a high gradient established the evaporation equilibrium maintaining a high diffusion gradient. Hence, from the previous Section 3.2.1, Section 3.2.2 and Section 3.2.3, we could see that airflow enhances the positive correlation between drying rate and sweat volume. Therefore, we could infer that the airflow has a similar positive, but to a lesser degree, effect on increasing the drying rate.

A summative regression analysis was done based on the previous discussions. As independent variables, evaporative resistance, sweat volume, and airflow were selected for the drying rate performance evaluation. The regression coefficients were found to be -2.059, 1.077, and 4.007 for evaporative resistance, airspeed, and sweat volume, with a coefficient of determination, R^2^ = 0.86 (Table 5). Considering the presence of a water-resistant, breathable fabric in the samples, such an R^2^ value can be identified as a good strength of the considered regression model. *p*-values for the coefficients were <0.05. Additionally, corresponding confidence intervals does not include 0, indicating the significance of such coefficients. Hence, the effects of such parameters were significant. The model shows that the evaporative resistance is negatively related and both sweat volume and air speed positively affect the drying rate performance. The impact of sweat volume is higher than other parameters.

For other fabric structural parameters, aside from the fabric composition, no significant attribute could be selected. Additionally, no significant trend was observed based on the moisture management properties. This could be due to the presence of a water-resistant, breathable fabric in the sample. Wetted radius could be significantly related to the drying rate performance. However, MMT measured the max wetted radius based on the sensor area. This means that such testing equipment could not address the actual sweat-wetting area. In summary, MMT parameters are ineffective in addressing fabric drying rate; this is more evident for coated, laminated fabrics. However, the temperature-time graphs’ shape for the fabric drying is closely related to the MMT parameters. Hence, it can be inferred that MMT parameters are more applicable to addressing the temperature changes of the drying fabrics.

## 4. Theoretical Analysis of the Sweat Drying of Protective Fabrics

**Figure 8:**
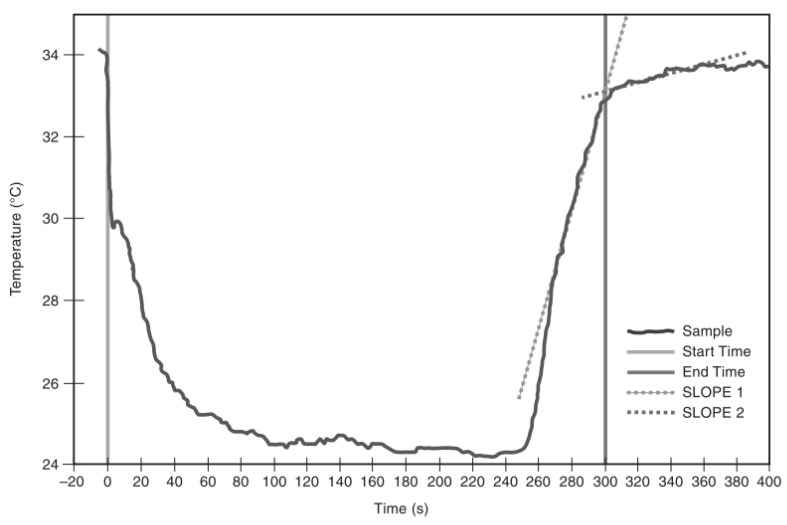
Temperature vs. Time curve to determine drying time [36].

Moisture may transfer through the textile materials in vapor and liquid form [32]. In the general wetting and drying curve (Figure 8) of temperature time plotting demonstrated in AATCC 201 standard for drying rate tester described in Figure 1, the temperature falls immediately at the point of water application [27,36]. At the point of water application, the surface temperature of the fabric coming below the ambient temperature (34 °C) is due to the water absorption. However, there is an initial slight increase in temperature (at around 30 °C) which subsequently declines. The initial slight increase is due to the releases of absorption heat. The temperature of the fabric then decreases again. This phase can be identified as ‘Declining temperature phase’ or ‘Increasing drying rate’ phase. After reaching a certain point in the temperature time plotting, fabric holds this lowered temperature for a long time before increasing back to ambient temperature. Fabric holds this lower temperature due to achieving the dynamic balance of heat. At this stage, heat loss due to the evaporation is equal to the heat gain by absorption, radiation, and convection from surrounding [27]. This is the stage of ‘Constant drying rate’ or ‘Constant temperature phase’, where the fabric temperature is constant theoretically. As mentioned earlier, the unbound water molecules are mostly evaporated from the fiber surface in this stage.

In the later stage, fabric temperature increases again to environmental temperature where heat release due to evaporation is lower than the heat gain by fabric from absorption of vapor around the micro-climate, radiation, and convection. In this stage, the bounded water molecules start to evaporate and fabric’s temperature rises rapidly [27]. This phase can be identified as ‘Decreasing drying rate’ or ‘Increasing temperature rate’ phase. In this stage, evaporation front moves into the fabric [34]. Therefore, drying process involves simultaneous heat and mass transfer between to or from the surface, and within materials, the motion of the particles inside the material, mechanism of moisture migration in the solid material [37]. Absorption of textile materials is theoretically a process of wetting, wicking and vapor transmission [38] and drying of textile materials is a very energy-intensive process and is a very broad subject [37].

### 4.1. Wetting and Absorption

**Figure 9:**
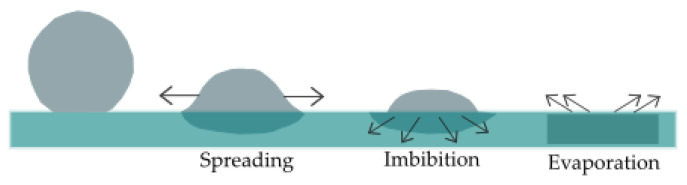
Spreading, imbibition and evaporation of droplet on porous substrates [49].

Absorption and transportation of the liquid is one of the crucial aspects of the fabrics used in performance clothing. Moisture in clothing has been found to be the most significant factor which contribute to discomfort [39,40]. Moisture present in the clothing increases the friction between skin and fabric, which causes a clingy sensation and eventually causes an increase in fatigue felt by the wearer [39,41]. For the clothing worn under very high temperatures, this problem is even more serious [39]. Excellent water absorption and transportation properties of the fabrics is potentially able to minimize the wetness feel on skin, accelerate the sweat drying and ensure comfort [39]. The transfer of liquid within the fabrics happen in two steps; wetting and subsequent wicking [32]. When a fabric comes into contact with liquid, wetting is the initial behavior shown by the fabric [42]. Wetting causes the capillary pressure and liquid reaches to the spaces of the clothing [43]. Maintaining the capillary flow is what defines wicking [42], which eventually further increases the spreading of the liquid or vapor throughout the fabric by the evaporation of moisture (Figure 9) [43]. Wetting is a required/pre-requisite of wicking; a liquid which does not wet the fiber cannot wick the fiber [39]. The interaction between the cohesion (within the liquid) and adhesion (between the solid and liquid) forces determines whether the wetting will take place and also the spreading of the liquid over the surface [39]. Wicking is determined by the arrangement of the fibers and yarns in the fabric [44], whereas, wetting is decided by the surface properties of the fiber and liquid [44]. Interaction of liquid with textiles are as follows; fiber surfaces are wetted by filling the pores and rough pits along the surface, liquid transportation through pores, capillaries and cavities in the materials, adsorption and migration along the surface, and diffusion into the fiber interior [45,46,47]. Absorption of the fiber (porous medium) depends on the liquid properties, geometry of the pores, and the interaction between the liquid and fiber [48]. Goncalves et al. studied the droplet evaporation on the porous media and found that wicking extends the surface area of wetting and thus promotes the evaporation in porous substrates [49].

The reason for water moving in a porous media is known as water activity a_w._ This is the measure of the energy of the water in a specific system. Water activity a_w_ is the driving force of water movement from the high activity region to low activity region. Several factors control the value of a_w_ in a system, which has been summarized mathematically in Kelvin equation [38].

(2) $$a_{w}=\frac{\rho_{v}}{\rho_{\mathrm{sv}}}=e^{\frac{-2\gamma M}{r\rho \mathrm{RT}}}$$

where γ = surface tension, M = molecular weight of water, ρ = density of water, T = absolute temperature, r = capillary radius.

Depending on the presence of water attracting group in the fiber, the water molecules can or cannot be directly attached. The water molecules absorbed by the hydrophilic groups are known as directly attached water. Once all the hydrophilic groups have absorbed water molecules, further layers on the top of the water molecules already absorbed may be formed which is known as indirectly attached molecules [38]. These directly and indirectly attached water molecules are also known as bounded and unbounded water molecules.

### 4.2. Evaporation and Drying

Studies have found two groups of factors that affect the drying behavior of fabrics; material properties and factors that control the heat and moisture transfer to and from the fabrics [29,50]. Studies have also found that drying rate happens in two different rates: constant and declining drying rate. There is also initial ‘increasing drying rate’ for a short time. This phase is also the called ‘wetting phase’ or ‘declining temperature phase’. We have considered this phase as ‘Increasing drying rate’. Though most of the literature skipped this stage, we considered the initial ‘declining temperature phase’/ ‘increasing drying rate’ phase as one of the drying steps. Therefore, we said drying occurs in three different steps, (i) ‘Increasing drying rate’, (ii) ‘constant drying rate’ and (iii) ‘decreasing drying rate’. In the ‘constant drying rate’ phase, the diffusion rate of the water to the surface exceeds the rate of evaporation [29,51]. Mass transfer rates and external heat between the water and fiber control the drying rate during this period. The rate of drying falls off after a “critical moisture content”. At this period, the rate of evaporation exceeds the rate of diffusion from the fiber interior to surface. The rate of drying in this period is controlled by the transfer of water through the fabric [29,51]. Water evaporation is a process where water molecules completely break the hydrogen bonds with other molecules located in the interface of water-air, and then move to gas phase region [52]. Sweat moisture transfer which takes place while air is flowing over the moisture layer is known as convection process. This process is known as forced convection process. The vapor concentration difference between the fabric surface and environment determines the amount of moisture or mass flow through evaporation. Therefore, in windy or air flow conditions, the convection process plays important role in transferring moisture from skin to fabric and then to atmosphere [32].

Drying equations in constant-rate and falling-rate periods were defined by Stelle (1959) [53].

Constant drying rate period,

(3) $$\frac{\mathrm{dW}}{\mathrm{dt}}=\mathrm{hA}\frac{\Delta T}{\lambda }=\mathrm{KA}\;\Delta P$$

Declining drying rate period,

(4) $$\ln\frac{W}{W_{\mathrm{crit}}}=\mathrm{Kt}$$

W = water content of the fabric (g/cm^2^), W_crit_ = critical moisture content of fabric (g/cm^2^), t = drying time in minute, h = total heat transfer coefficient (W/(m^2^K)), A = area (cm^2^), λ = latent heat of vaporization of water at drying temperature (kJ/kg), K = mass transfer coefficient (m/s), ΔT = T_a_ − T_s_, where T_a_ = temperature of air, T_s_ = temperature of drying surface (°C), ΔP = P_s_ − P_a_, where P_s_ = vapor pressure of water at the surface temperature, P_a_ = partial pressure of water in the air (Pa). Gonçalves et al. [49] described the evaporation rates as

−dm/dt = 4 β D (1 − H) c_v_ (r_w_ − r_d_) (5)

where, radius of droplet is r_d_, the wetted radius is r_w_, D is the diffusivity of the water vapor, H is relative humidity, c_v_ is the saturated water vapor concentration. β = 1.08, which is the calibration factor for the fabric gives better fit for the slope.

## 5. Conclusions

This paper investigated the sweat drying performance of fire protective fabrics used in high-risk sector workers’ clothing. In contact with the sweat moisture, the fabric gets wet. The wetting property of textile materials is determined by the fiber and fabric coating. Wicking, the subsequent process of wetting, depends on the arrangement of fibers and yarns in the fabric. Wetting and wicking are then followed by the drying process. Simultaneous heat and mass transfer within the textile materials and between to or from the surface of the materials is involved in the drying process. Three distinctive phases of the sweat drying process can be identified: increasing, constant, and declining drying rate. Fabrics’ moisture management properties can be related to their temperature change in those phases. Fabrics’ liquid moisture transport to the evaporation surface determines the duration of the increasing rate phase. Increased sweat volume enhances the sweat drying performance in the constant rate phase due to having more wetted area in the evaporation front. Evaporative resistance further increased the drying rates in the ‘declining drying rate’ phase. Moreover, evaporative resistance of fabrics has been found to significantly determine the sweat drying rate of different types of fabrics, regardless of the presence of the coating. The hygroscopic portion in the fabric composition will enhance the drying rate further. The effect of airflow on sweat drying performance is significant, but not more than increased sweat-wetted area in the evaporation front. Addressing the sweat drying performance and the corresponding temperature change in the three distinct phases could facilitate a better understanding of the comfort properties of high-risk sectors’ work gear. This research explored the sweat-drying performance of high-risk sector workers’ gear to address the thermoregulation performance of high-risk sector work-clothing through sweat evaporation. It has been observed that evaporative resistance, wetting performance of the clothing material, and their hygroscopic/hydrophobic polymeric compositions have significant effects on the sweat drying performance as well as subsequent thermal comfort of the high-risk sector workers. The findings of this research will advance the field by developing knowledge on providing better comfort and safety to high-risk sectors’ workers.

This research identifies the parameters affected the sweat drying performance through experimental and theoretical analysis; however, this study did not develop any model for predicting the sweat drying performance. In future, a more controlled experimental studies could be conducted to develop the model for predicting the sweat drying performance of fabrics. This could lead towards the development of state-of-the-art textile fabrics for protective clothing in order to provide better comfort and safety to high-risk sector workers worldwide.

## Figures and Tables

**Table 1:** Details of the selected fabrics.

Fabric Samples	Fiber Composition	Fabric Construction	Physical Properties				
			Thickness ^a^ (mm)	Weight ^b^ (g/m^2^)	Air Permeability ^c^ (cm^3^/cm^2^/s)	Thermal Resistance ^d^ (C × m^2^/W)	Evaporative Resistance (Pa × m^2^/W) ^d^
a	99% Meta Aramid 1% Carbon Fiber	66 × 60	0.579	148	6.058	0.022	2.653
b	50% Meta and 50% Para aramid	85 × 58	0.483	272	13.580	0.007	2.591
c	50% Meta and 50% para-aramid	72 × 52	0.488	204	33.420	0.021	4.026
d	60% Meta aramid 40% Cotton	75 × 55	0.467	237	47.840	0.021	2.453

^a^ Measured according to ASTM D 1777 under 1 kPa pressure; ^b^ Measured according to ASTM D 3776; ^c^ Measured according to ASTM D 737 under air pressure differential 125 Pa; ^d^ Measured according to ASTM D 737.

**Table 2:** The moisture management property of the fabrics.

Fabric Samples		Moisture Management Property					
		WT (s)	AR (%/s)	MWR (mm)	SS (mm/s)	AOTC (%)	OMMC
a	Top	6.178	168.128	5	0.889	−865.541	0.002
	Bottom	70.247	7.477	5	0.073		
b	Top	2.942	75.218	25	4.527	61.336	0.572
	Bottom	3.092	81.328	25	4.282		
c	Top	3.894	15.826	14	1.550	745.804	0.579
	Bottom	2.901	7.330	12	1.927		
d	Top	3.295	79.361	25	5.238	230.780	0.734
	Bottom	3.220	71.832	25	4.930		

**Table 3:** Sweat drying performance of the selected fabrics as per AATCC 201 standard.

Sample	Volume (mL)	Drying Time (s)	Drying Rate (mL/h)	Average Drying Rate
a	0.200	507.9	1.42	1.42
	0.200	509.3	1.41	
	0.200	502.9	1.43	
b	0.200	555.2	1.30	1.31
	0.200	549.7	1.31	
	0.200	545.5	1.32	
c	0.200	1241.1	0.58	0.59
	0.200	1220.7	0.59	
	0.200	1184.3	0.61	
d	0.200	526.7	1.37	1.37
	0.200	537.5	1.34	
	0.200	512.9	1.40	

**Table 4:** Sweat drying performance of the selected fabrics under different ambient conditions.

Fabric Samples	Ambient Air Speed (m/s)								
	0.5			1			1.5		
	SV 0.5 mL	SV 1 mL	SV 1.5 mL	SV 0.5 mL	SV 1 mL	SV 1.5 mL	SV 0.5 mL	SV 1 mL	SV 1.5 mL
a	2.2	4.22	5.44	2.74	4.92	6.23	2.27	5.06	7.12
b	2.11	3.75	4.96	2.9	4.52	6.25	3.2	4.98	8.03
c	0.83	1.04	2.67	0.72	2.1	2.56	1.13	2.49	3.44
d	2.98	5.34	7.45	3.66	6.53	9.1	3.1	6.13	10.39

**Table 5:** Regression model of fabric sweat drying rate.

Regression Parameters	Coefficients	Standard Error	*t* Stat	*p*-Value	Lower 95%	Upper 95%	R Square
evaporative resistance	−2.059	0.232	−8.867	1.393 × 10^−10^	−2.530	−1.588	0.863 (adjusted R square = 0.851)
air speed	1.076	0.363	2.970	0.005	0.341	1.812	
sweat volume	4.007	0.330	12.128	2.827 × 10^−14^	3.337	4.678	

## Data Availability

The data presented in this study are available on request from the corresponding author.
